# Nonprescribed use of tranquilizers or sedatives by adolescents: a Brazilian national survey

**DOI:** 10.1186/1471-2458-13-499

**Published:** 2013-05-24

**Authors:** Emerita S Opaleye, Ana R Noto, Zila M Sanchez, Tatiana C Amato, Danilo P Locatelli, Michael Gossop, Cleusa P Ferri

**Affiliations:** 1 Departamento de Psicobiologia, Universidade Federal de São Paulo, Rua Botucatu, 862- 1° andar, São Paulo CEP 04023-062, Brasil; 2 King’s College London, Institute of Psychiatry, 4 Windsor Walk, London SE5 8AF, UK

## Abstract

**Background:**

Although the nonprescribed use of tranquilizers or sedatives by adolescents is a cause for concern in many countries, there is a shortage of data from low and middle income countries (LAMIC). The present study aims to estimate the prevalence of nonprescribed use of tranquilizers/sedatives by adolescents in Brazil, and to assess how socioeconomic and demographic circumstances, as well as indicators of access to these substances are associated with their use and with risk perception.

**Methods:**

A cross-sectional study was conducted using a multi-stage probability sample of 18131 high school students from public and private schools from all 27 Brazilian state capitals. A self-reporting questionnaire was used to obtain information on social and economic circumstances, nonprescribed use of tranquilizers or sedatives and risk perception of their use.

**Results:**

Lifetime nonprescribed use of tranquilizers or sedatives was reported by 5% of respondents, more commonly among females (OR: 2.19, 95% CI: 1.75-2.75) and those attending private schools (OR: 1.47, 95% CI: 1.17-1.84). The use of tranquilizers/sedatives by relatives or friends was associated with nonprescribed use by the participant (OR: 4.26, 95% CI: 3.46-5.23) and a majority of lifetime users obtained these substances from a family environment (82%). Previous medical prescription was independently associated with nonprescribed use (OR: 6.61, 95% CI: 4.87-8.98) and with low risk perception (OR: 2.42, 95% CI: 1.12-5.24).

**Conclusions:**

A substantial proportion of Brazilian adolescents use nonprescribed tranquilizers/sedatives. Easy access to these substances seems to play an important role in this use and should be tackled by preventive and treatment interventions.

## Background

The abuse of nonprescribed drugs among young people appears to be a growing global problem. Among United States youth, the use of prescription drugs exceed that of illicit drugs, excluding cannabis [1]. A survey in the USA showed that 6.5% of students had used nonprescribed tranquilizers [2] whilst a study in 31 European countries showed an average prevalence of 5.6% of those who had ever used tranquilizer/sedatives with considerable variation between countries (from around 1.5% in Ukraine and the United Kingdom to about 13% in Lithuania and France) [3].

These substances may be seen as being “safer” than illicit drugs since they are produced by pharmaceutical companies, prescribed by physicians and widely consumed by millions of people [4,5]. Their widespread availability and pharmacological properties make them particularly susceptible to abuse and dependence [6,7]. Evidence indicates that early use of psychotropic substances is likely to lead to higher levels of dependence later in life along with the related health and social problems [8]. It is therefore particularly important to have some better idea about their use among adolescents.

Although the misuse of prescription drugs is increasing rapidly, data about nonprescribed use of prescription drugs are not systematically collected in most countries, especially in Low and Middle Income Countries (LAMIC), where policies for drug control are more likely to fail. Being the largest country and most important economy of Latin America, Brazil represents a huge market for the pharmaceutical industry, where BZD are among the most widely prescribed drugs [9,10].

This study looks at the use of nonprescribed tranquilizers or sedatives among a sample of Brazilian high school students. It is hoped that studies with Brazilian adolescents can make a substantial contribution to the knowledge gap about nonprescribed use of tranquilizers by adolescents in LAMIC. The study used data from a cross-sectional survey representative of high school students (n = 18131) from all 27 Brazilian state capitals to estimate the prevalence and describe the nonprescribed use of tranquilizers/sedatives among Brazilian adolescents and to assess how sociodemographics and economic circumstances, as well as indicators of access to tranquilizers/sedatives are associated with their misuse.

## Methods

### Participants/settings

A cross-sectional survey was conducted with a sample of students attending private and public schools from all the 27 Brazilian state capitals between March and October 2010.

The study’s target population was designed as a representative sample of high school students (10th to 12th grade), with a two step random selection process. A list of all private and public schools for each city included in the study was obtained by the means of a national governmental register. The schools were then stratified according to the grade levels (middle schools only, high schools only, and both levels). Based on a proportional size sample, allocated according to the number of students for each stratum, a randomized process selected an average of 3 clusters (classroom) in each school. All the students in the selected classroom were then invited to participate in the survey and to answer the self-report questionnaire. These methods have been previously described elsewhere [16].

A total of 789 schools participated in this study, with a school response rate of 86%. The student response rate was 79.2% (20.5% were absent on the day of survey and there was 0.3% refusal rate) with a total of 19230 questionnaires completed. We included a question for a fictitious drug and 98 students attending high school who answered it positively were excluded. The present study is limited to high school students aged between 13 and 19 years old (n = 18131).

### Procedures

The questionnaires were self-administered and were completed inside the classroom without the presence of any school staff and under the supervision of a team of trained interviewers (all interviewers received 3 full days of training). It took students 40 minutes on average to complete the questionnaire. To avoid contamination the survey was conducted in a single visit to each school. All procedures were standardized and applied in the same way in each of the schools.

### Ethics

Students were informed about the aims of the study and were reassured that their participation was voluntary, that they could choose not to participate at any time, and that all the information provided was anonymous and to be used for research purposes only. Written informed consent was obtained from the principal of schools. The study was approved by the Research Ethics Committee of the Federal University of São Paulo.

### Measures

Our pencil and paper questionnaire was based on a World Health Organization instrument [14] and on the European School Survey Project on Alcohol and Other Drugs (ESPAD) questionnaire [15] which are specific for self-report surveys about substance use among adolescents attending school and have been previously used in the Brazilian population [16].

### Sociodemographic and economic circumstances

We obtained information on gender, age and type of school (public or private). Socioeconomic status (SES) was determined using a standard scale [17], which considers the educational level of the head of the household, possession of household assets (such as television, DVD players, cars, refrigerators etc) and number of housekeepers (such as driver, cleaner, gardener etc). This scale categorizes participants into 5 social classes (from A to E, where A is the highest and E the lowest).

### Nonprescribed use of tranquilizers/sedatives

We limited our questions to the use of tranquilizers/sedatives without a medical prescription. We obtained information on lifetime use with the question: “Have you ever taken a tranquilizer or sedative without a medical prescription? E.g.: Diazepam, Dienpax®, Valium®, Lorax®, Rohypnol®, Psicosedin®, Somalium®, Apraz®, Rivotril®, Alprazolam, Lexotan®, Dalmadorm®, Dormonid®, Bromazepam, Frontal®, Olcadil® (DO NOT CONSIDER TEA OR NATURAL MEDICINES)”. Similar questions were asked regarding past year and past month use. We also asked about the frequency that the substance was consumed in the past month, the age of first use and about the reasons for using tranquilizers or sedatives.

### Indicators of access to tranquilizers/sedatives

All participants were asked about having received a medical prescription for tranquilizers or sedatives and about the use of these substances by a relative (parents, step-parents or siblings) or a close friend. Tranquilizers or sedatives users were asked how they obtained the medication with the following possible answers: “from a relative”, “from a friend”, “I get it from my home” and “other”.

### Risk perception of nonprescribed use of tranquilizers/sedatives

Students were asked about their risk perception of regular misuse of tranquilizers or sedatives. Responses ranged from “no”, “mild”, “moderate” “severe” risk and “I don’t know the risk”. These were further categorized into “low risk” (no risk plus mild risk) and “high risk” (moderate plus severe risk).

### Data analyses

All analyses were performed in STATA Version 11 weighted to correct for unequal probability of selection in the sample. The complex survey design took into account the stratum (city and type of school), the conglomerate (school as primary sampling unit), the sampling weight and the probability of selecting the student who answered the questionnaire.

Logistic regression was used to estimate the crude and adjusted Odds Ratios of the association between all sociodemographic characteristics, indicators of access to tranquilizers/sedatives and student’s risk perception with their lifetime use. Logistic regression models were also used to estimate the association between having received a prescription for a tranquilizer or a sedative in the past and their perception of risk related to their misuse (adjusted for age, gender, social class, type of school and tranquilizers or sedatives use by a family member and/or friends). The latter analysis was limited to students reporting lifetime use of these substances (n = 1026). We excluded 94 students who said “do not know” for risk perception. Results are presented via weighted proportions (wgt%), crude Odds Ratios (cORs), adjusted Odds Ratios (aORs) and 95% confidence interval.

## Results

Table 1 describes the sample (n = 18131) and the lifetime of nonprescribed tranquilizers users (n = 1076). Just over half of the participants were female (55.3%), about two-thirds were older adolescents (between 16 and 19 years old), and the majority attended public schools (78.6%). Thirty-six per cent of participants were in the lowest social classes (C-E). 

**Table 1:** Sample characteristics, prevalence and correlates of nonprescribed lifetime use of tranquilizers or sedatives among Brazilian adolescents (n = 18,131)

	**Total (n = 18131)**	**Lifetime use prevalence (n = 1076)**	**Crude OR (95% CI)**	Adjusted OR* (95% CI)	**p value**
Age (years)	wgt%	wgt% (95% CI)			
13-15	35.6	4.5 (3.9-5.2)	1.00	1.00	
16-19	64.4	5.3 (4.8-6.0)	1.18 (0.98-1.41)	1.37 (1.14-1.66)	0.001
Gender					
Male	44.7	3.1 (2.6-3.6)	1.00	1.00	
Female	55.3	6.6 (6.0-7.3)	2.22 (1.83-2.70)	2.19 (1.75-2.75)	<0.001
SES					
(lowest) C-E	36.0	3.7 (3.2-4.4)	1.00	1.00	
B	35.3	5.8 (5.1-6.6)	1.58 (1.27-1.96)	1.36 (1.06-1.75)	0.015
(highest) A	10.4	9.3 (8.0-10.8)	2.61 (2.07-3.30)	1.77 (1.27-2.48)	0.001
Not reported	18.4	3.6 (2.7-4.7)	0.95 (0.69-1.31)	1.06 (0.76-1.49)	0.734
Type of school					
Public	78.6	4.2 (3.7-4.7)	1.00	1.00	
Private	21.4	8.1 (7.4-8.9)	2.02 (1.71-2.38)	1.47 (1.17-1.84)	0.001
Use of tranquilizers or sedatives by family members or friends					
No	90.1	3.8 (3.4-4.2)	1.00	1.00	
Yes	10.0	17.5 (15.3-20.0)	5.43 (4.50-6.54)	4.26 (3.46-5.23)	<0.001
Received a prescription of tranquilizers or sedatives					
No	90.0	3.6 (3.3-4.1)	1.00	1.00	
Yes	2.6	28.8 (23.1-35.1)	10.66 (7.98-14.22)	6.61 (4.87-8.98)	<0.001
Do not remember	7.4	13.5 (11.5-15.7)	4.11 (3.28-5.15)	3.58 (2.77-4.63)	<0.001
Risk perception of nonprescribed tranquilizers or sedatives use					
High	81.3	4.9 (4.4-5.4)	1.00	1.00	
Low	7.2	8.4 (6.5-10.8)	1.79 (1.33-2.40)	1.53 (1.09-2.15)	0.015
Do not know	11.5	4.1 (3.1-5.5)	0.84 (0.61-1.16)	1.07 (0.76-1.50)	0.692

* Adjusted by all variables in the table.

Lifetime nonprescribed use of tranquilizers or sedatives was reported by 5.0% (95% CI: 4.6%-5.5%) of respondents (n = 1076). One in 10 participants reported the use of tranquilizers or sedatives by a family member or a friend, 2.6% had received a prescription for these substances in the past and 7.2% perceived their regular use as low risk. Although the majority of participants did not report use of tranquilizers or sedatives by a relative or friend and had never received a medical prescription for these substances, the nonprescribed use of tranquilizers or sedatives was more common among those who responded positively to these two questions (17.5% and 28.8% respectively).

We estimated the association between lifetime nonprescribed use of tranquilizers or sedatives and sociodemographic characteristics (age, gender, SES, type of school); indicators of access to tranquilizers or sedatives (use by family/friends and having received a medical prescription in the past) and risk perception of nonprescribed use (Table 1). Adjusted analysis showed that females (adjusted OR = 2.19; 95% CI: 1.75-2.75) and older adolescents (adjusted OR = 1.37; 95% I 1.14-1.66) were more likely to report lifetime nonprescribed use of tranquilizers or sedatives. Wealthier students (from private schools and from the highest social class) were more likely to report lifetime nonprescribed use of tranquilizers or sedatives. Adolescents whose relatives or friends use these substances were more likely to report misuse compared with those who did not report such use by family or friends (OR = 4.26; 95% CI: 3.46-5.23). Having received a medical prescription for tranquilizers or sedatives in the past increased by 6.61-fold (95% CI: 4.87-8.98) the odds of a participant to report nonprescribed use. Compared to those reporting high risk perception, participants with low risk perception were also more likely to report lifetime nonprescribed use.

Table 2 describes the pattern of consumption among those who reported having ever used nonprescribed tranquilizers or sedatives (n = 1076). Sixty six percent of them (n = 703) reported nonprescribed use in the past year (3.3% of the total sample) and nearly half of those who used in the previous year reported misuse in the last month (n = 303, 30% of lifetime users).

**Table 2:** Nonprescribed use of tranquilizers or sedatives by ever users (n = 1076)

	**wgt% (95% CI)**
Past year use	
	66.6 (62.6-70.4)
Past month	
	30.0 (26.4-33.8)
Frequency of recent use (past month)	
None	70.0 (66.1-73.6)
1 to 5 days	24.2 (21.2-27.4)
6 to 19 days	3.1 (2.0-5.0)
20 or more days	2.7 (1.5-4.9)
Reasons for use	
To sleep/sleep better	47.9 (43.5-52.4)
To better deal with my anxiety	47.7 (43.5-51.9)
To get high	4.4 (2.8-6.7)
Sources for the first use	
Given by a relative	52.7 (48.0-57.4)
Given by a friend	11.0 (8.0-15.0)
Available in the household	29.3 (25.5-33.3)
Others*	7.0 (4.5-10.6)

* Bought from drugstores without prescription, from drug dealers or used false prescription.

Among those who reported past-month use, most reported having used tranquilizers or sedatives between 1-5 days. A great majority (n = 854) of ever users (n = 1076) reported self-medication as a reason for their nonprescribed use (47.7% to relieve anxiety symptoms and 47.9% to help sleeping) and only 4.4% reported taking tranquilizers or sedatives to get high. Most of those reporting lifetime use (52.7%) obtained tranquilizers or sedatives from a relative, and 29.3% found it available in the household. More than half of the students (57%) who reported using tranquilizers or sedatives to get high said that they had obtained the medication from friends and a quarter had had their first use before they were 14 years (data not shown in the tables).

The association of having received a prescription in the past with risk perception regarding regular nonprescribed use was also estimated (adjusted by age, gender, social class, type of school and use by family member and/or friends). Those who received a prescription for tranquilizers or sedatives in the past were more likely to perceive as low the risk associated with regular nonprescribed use (OR: 2.42, 95% CI: 1.12-5.24).

## Discussion

In this sample of 18131 students from Brazilian private and public schools, 5% reported lifetime use of nonprescribed use of tranquilizers or sedatives, and 3.3% had used them in the past year. Prevalence was twice as high in females as males and more common in wealthier participants. Easy access to tranquilizers or sedatives (use by family member and friends and having previously received a prescription) was associated with this misuse and the substance was commonly obtained within their own home (given by a relative, available in the household). Despite nonprescribed use most reported using to relieve anxiety symptoms and to sleep.

Prevalence of lifetime use of nonprescribed tranquilizers or sedatives by Brazilian students was very similar to that reported among students in Europe (5.6%) [3] and the USA (6.3%) [2]. Nationally representative samples of adolescents in the USA show lifetime prevalence of sedatives to be less than 4% [18] and 2% of past-year use of tranquilizers [18] which is lower than that found among Brazilian students (3.3%). A previous survey conducted in Brazil in 2004 and restricted to public schools [19] found a prevalence of 4.1% among students aged 15 years and above, which is slightly lower than that found in our study.

Poverty is linked to poorer health behaviour and health in general, including mental disorders [20,21]. Depression and substance misuse are important examples of this disadvantaged cycle. Studies conducted with nonprescribed use of prescription drugs have also shown higher prevalence of their use among people in more disadvantaged socioeconomic circumstances [18]. Our findings however indicated the opposite; showing that nonprescribed use of tranquilizers/sedatives was more common among those in higher social classes and those attending private schools. People in conditions of economic hardship in LAMIC such as Brazil have less access to health services, and therefore they and their acquaintances are less likely to have access to any kind of medicine.

It has generally been reported that nonprescribed drug use is more common among females compared to males, especially with regard to tranquilizers and sedatives [22]. The higher prevalence of nonprescribed use of tranquilizers or sedatives among females found in our study corroborates studies in this aspect [3,23].

Moreover, in our study those who had previously received a medical prescription for tranquilizers or sedatives were more likely to do a nonprescribed use of these substances. A study conducted in the USA [24] found that individuals aged 18 and over with a medical prescription for anxiety were 61% more likely to use nonprescribed anti-anxiety drugs compared to those who did not receive a prescription. They were also more likely to report abuse and/or dependence, even after adjusting for sociodemographic variables and anxiety severity. Receiving a prescription for a tranquilizer or a sedative may encourage adolescents to use them again when they face similar symptoms for which they received a prescription. However, due to the nature of this study, we cannot establish the direction of effect: indeed, nonprescribed use may influence adolescents to seek a medical prescription to maintain their use.

In our study most adolescents (more than 80%) who reported nonprescribed use of tranquilizers or sedatives had obtained these in their household environment. On the other hand, it is possible that friends are more likely to divert their prescription drugs for the purpose of sensation-seeking. A high proportion of adolescents in this sample who reported nonprescribed use for sensation-seeking stated obtaining the medicine from friends.

A medical prescription is legally required to purchase tranquilizers and sedatives in Brazil for all age groups, but because of failure in the law enforcement, purchasing these substances without a prescription is an established problem in Brazil [9,25]. However this is likely to be more difficult for adolescents, because of their age. In our study only a small proportion stated spontaneously that they had purchased the medication without a prescription.

Perceiving the use of a substance as no or low risk has been found to be associated with their misuse [26], and this was also found in our study for tranquilizers and sedatives. However, little is known about the factors associated with risk perception of tranquilizers or sedatives use. Our findings showed that among those who were users of nonprescribed tranquilizers or sedatives, having previously received a medical prescription was associated with low risk perception, even after adjusting for their use by family or friends; this suggests that receiving a medical prescription may have a stronger influence on adolescents’ beliefs about the risks involved in the nonprescribed use of tranquilizers or sedatives compared to their relatives’ behaviour regarding use of these substances.

Participants in this study represent students attending private and public schools from the 27 Brazilian state capitals and therefore caution is required when generalizing our findings to students from the whole country and most importantly, to students attending schools in rural areas. However, we have a large sample size which is representative of students from the biggest cities in Brazil from all the country’s regions. Most studies on this subject have been conducted in developed countries and our findings add important information from a LAMIC perspective. Another limitation is that we can not extrapolate these findings to those students who were absent on the day of the survey or to adolescents who are not attending school in Brazil: some adolescents who are involved in substance abuse are less likely to be regular school attendees.

Self-report paper and pencil technique might lead to over or under-estimation of true substance misuse prevalence. To minimize the overestimation possibility we asked about the use of a ‘fictitious’ drug and those who answered positively to this question were excluded from the sample. Despite this potential limitation, anonymous self-report surveys are a very cost-effective method to investigate substance use from an epidemiological perspective [27]. Our sample was enriched by a high response rate, since almost all of the students who were invited to participate in our study, agreed to do so.

## Conclusions

The results of this study showed that availability, low risk perception and having received a medical prescription in the past are important correlates of nonprescribed use of tranquilizers or sedatives among Brazilian students. These findings, together with the high prevalence we found, are relevant for policy makers in seeking to develop strategies to prevent this behaviour, such as improving medical education about the need for caution in initiating tranquilizers or sedatives prescriptions in general, and particularly for adolescents. Also, educational programmes developed to prevent nonprescribed use of tranquilizers or sedatives among adolescents should include information for adolescents and their families regarding the risks associated with the misuse of these substances.

In addition, doctors should inform their adult patients that a prescription is for the named individual only and the patient should be told of the risks of diverting the prescription or of making tranquilizers and sedatives available to other members of the family. Pharmacists can also have an important role in preventing nonprescribed use of these substances, not only advising users about exclusivity of prescription, but also about the care that should be taken regarding storage and disposal of medicines at home.

## Abbreviations

LAMIC: Low and middle income countries; INEP: National institute on educational research and studies (Portuguese abbreviation to Instituto Nacional de Estudos e Pesquisas Educacionais); ESPAD: European school survey project on alcohol and other drugs; SES: Socioeconomic status.

## Competing interests

The authors declare that they have no competing interests.

## Authors’ contributions

EO, AN, ZS, TA and DL participated on conception of the original study and data collection. All authors participated in the conception of this specific analysis. EO and CF conducted the statistical analysis. EO wrote the first draft of the manuscript under CF’s supervision. All authors revised and approved the final manuscript.

## Pre-publication history

The pre-publication history for this paper can be accessed here:

http://www.biomedcentral.com/1471-2458/13/499/prepub
